# Application Effect of Intra-Abdominal Pressure Monitoring System in Early Enteral Nutrition Nursing of ICU Patients

**DOI:** 10.1155/2022/3545278

**Published:** 2022-01-29

**Authors:** Meiling Song, Peiru Zhao, Wenjun Hu

**Affiliations:** ^1^Sandun Hospital Area 10 Ward, Zhejiang Hospital, Hangzhou, Zhejiang 310030, China; ^2^Lingyin Hospital Area 20 Ward, Zhejiang Hospital, Hangzhou, Zhejiang 310013, China; ^3^Sandun Hospital Area ICU, Zhejiang Hospital, Hangzhou, Zhejiang 310030, China

## Abstract

In order to study and explore the application effect of intra-abdominal pressure monitoring in guiding ICU patients to implement early enteral nutrition therapy, we selected patients admitted to the emergency department of a hospital and classified them into groups A (*n* = 105) and B (*n* = 98). Among them, the A group gave early enteral nutrition treatment by monitoring the residual gastric mass, and the B group gave early enteral nutrition treatment by monitoring the stomach. It has been established that, compared to gastric residue monitoring, intra-abdominal pressure monitoring in ICU patients with early enteral nutrition therapy has an obvious advantage, it helps to improve the prognosis of patients, and intra-abdominal pressure combined with gastric residual monitoring scheme can effectively reduce the early enteral nutrition in the ICU patients, the incidence of abdominal distension, vomiting, and make it reach the goal as soon as possible. Early enteral nutrition in patients with increased tolerance is of great significance.

## 1. Introduction

In recent years, the application of intra-abdominal pressure (IAP) monitoring technology in China has become increasingly widespread and has become one of the important physiological parameters for clinical diagnosis, disease treatment, and prognosis judgment [1]. Poor nutritional status is common in patients in intensive care unit (ICU) due to the rapid decline of their nutritional status, the incidence of acute gastrointestinal dysfunction is relatively high, and there are many reasons for it, but there is a lack of characteristic clinical manifestations in the early stage. Studies have confirmed that early enteral nutrition (EEN) supports; it can significantly reduce the incidence of serious gastrointestinal complications and promote the recovery of patients, so EEN needs to be started as soon as possible. According to reports, the probability of gastrointestinal intolerance during EEN via nasogastric tube is 56.3%, such as abdominal distension, diarrhea, and electrolyte imbalance [2]. In order to improve the tolerance of early enteral nutrition in ICU patients, more monitoring of gastric residual volume is used to clarify the tolerance of patients with EEN; however, it was found in clinical work that relying on the monitoring method of gastric residual volume, there are still many patients with complications such as abdominal distension and vomiting, which makes it impossible to clarify the time of enteral nutrition adjustment and improve patient tolerance [3]. Enteral nutrition is an important treatment for ICU patients. Enteral nutrition therapy should be carried out when gastrointestinal function allows and can be used safely. In order to evaluate the safety of enteral nutrition in clinical patients, it is often necessary to monitor gastric residue. However, whether to adjust the rate of enteral nutrition infusion according to the threshold of gastric residual volume is still controversial [4]. Early enteral nutrition support in critically ill patients plays an irreplaceable role in maintaining intestinal mucosal barrier and immune function. Studies have shown that early enteral nutrition can significantly reduce the incidence of serious gastrointestinal complications, promote patients' rehabilitation, and improve patients' quality of life. It is reported that the probability of gastrointestinal intolerance caused by intranasal nutrition is 56.3%, which seriously affects the prognosis of patients. The manifestations of gastrointestinal intolerance include gastrointestinal and metabolic complications, such as abdominal distension, diarrhea, and electrolyte disorder [5]. Therefore, in order to improve the tolerance of early enteral nutrition in ICU patients, studies have shown that Carron believes that people recognize that enteral nutrition meets the physiological needs of the human body; it is of great significance to maintain the intestinal mucosal barrier function, maintain the normal structure and function of the gastrointestinal tract, and prevent intrahepatic cholestasis [6]. Goenka and other studies have shown that IAP is significantly positively correlated with bladder pressure and intragastric pressure, and bladder pressure has a good correlation with IAP; it can objectively reflect the dynamic changes of IAP, diagnose ACS in time, and quickly evaluate its influence on organ function. The A-PACHEII scoring system is an objective, simple, and practical scoring method for evaluating the condition of critically ill patients and predicting the prognosis [7]. Chang and other studies have confirmed that the dynamic changes of IAP are related to the body's cardiopulmonary function and abdominal organ diseases; when IAP is continuously or repeatedly elevated, it can cause abdominal hypertension (IAH); subsequent progress may lead to abdominal compartment syndrome (ACS) characterized by increased airway pressure, hypoxemia, dyspnea, oliguria, and anuria; it causes a series of pathophysiological changes in the body, leading to multiple organ failure or obstacles and endangering the lives of patients [8].

In this study, dynamic intra-abdominal pressure monitoring was used to evaluate the gastrointestinal function of patients, so as to achieve early and reasonable enteral nutrition treatment and improve the prognosis of patients. The report is as follows.

## 2. Experimental Analysis

### 2.1. Materials and Methods

From January to December, 203 qualified patients who received early enteral nutrition therapy in Municipal People's Hospital were selected and divided into group A (=105) and group B (=98) by random number table method; using random number table method, they were divided into group A (*n* = 105) and group B (*n* = 98). Inclusion criteria: age >18 years old; the expected stay in ICU is >72 h; the mechanical ventilation time is expected to be >48 h; enteral nutrition can be accepted within 48 hours. Exclusion criteria: history of bladder surgery or neurogenic bladder, no indwelling catheter through the urethra; abdominal effusion; pregnant women. Comparing of gender, age, body mass index, acute physiology, and chronic health status scores between the two groups, the difference was not statistically significant (*P* > 0.05), and it was comparable. See Table 1.

### 2.2. Research Methods

Gastric residual volume monitoring early enteral nutrition group A patients was indwelled with a gastric tube, and gastric residual volume monitoring was performed before the start of enteral nutrition. The initial speed of the enteral nutrition solution was 20 ml/h, and the gastric residual volume was monitored every 6 h in patients with continuous enteral nutrition for 24 hours. The residual gastric volume is less than or equal to 200 ml, which can maintain the original speed; if the residual gastric volume is less than 100 ml, increase the enteral nutrition infusion rate by 20 ml every 6 hours until the target feeding rate; if gastric residual volume is >200 ml, suspend enteral nutrition and add gastrointestinal motility drugs; the residual gastric volume is monitored every 4 h. When the residual gastric volume is less than or equal to 200 ml, enteral nutrition is restarted [9].

Intra-abdominal pressure monitoring the intra-abdominal pressure of patients in group B was measured before the implementation of enteral nutrition; a controlled nutrient solution is pumped into a silicone rubber nasogastric tube at a constant rate for 24 hours; the daily target calories required by the patient are calculated at 25 kcal/(kg·d). Intra-abdominal pressure monitoring uses indirect cystometry. The measurement is carried out according to the standardized monitoring method of intra-abdominal pressure proposed by the World Association of Abdominal Cavity in 2013 [10].

### 2.3. Observation Indicators

Record and compare the incidence of early enteral nutrition intolerance, the proportion of patients who reach 80% of the estimated energy within 72 hours, mechanical ventilation time, ICU hospitalization time, incidence of ventilator-associated pneumonia, the incidence of multiple organ dysfunction syndrome, and the mortality rate during hospitalization. The occurrence of adverse reactions, including abdominal distension (a physical examination reveals that a part of the abdomen or the entire abdomen is swollen, full, and uncomfortable), vomiting (a large amount of stomach contents gushing from the mouth and nose, and the abdominal distension is relieved after vomiting), and diarrhea (the stool is thin and unshaped, accompanied by an increase in the frequency of bowel movements). Basic energy expenditure (basal energy expenditure (BEE)):

(1) $$\begin{array}{c} \text{man BEE}\left(\text{kcal}\right)=66.5+13.7\times W+5.0\times -6.8\times A,\\ \text{woman BEE}\left(\text{kcal}\right)=65.1+9.56\times W+1.8\times H-4.68\times A. \end{array}$$

*H*=height(cm) and *A*=age(years). Judgment of reaching the target feeding amount: the purpose is to achieve the nutritional needs of patients and maintain the energy required for basal metabolism. APECHEII score: the score distribution is 0 to 71 points; the higher the score, the worse the prognosis. record the indwelling time of the two groups of patients in ICU. Adjusting the time of enteral nutrition means that the nurse adjusts the time of the nutritional support plan within 3 days after the start of enteral nutrition [11, 12].

## 3. Results

**Figure 1:**
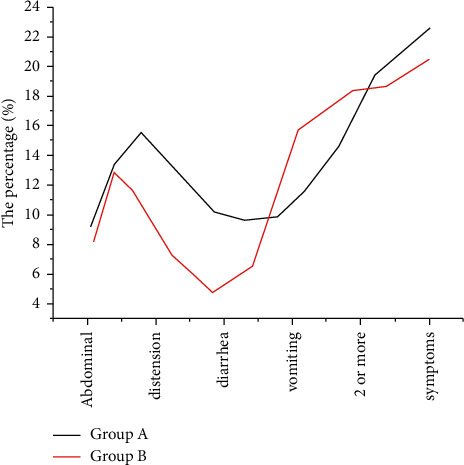
Comparison of the incidence of poor tolerance between the two groups.

The incidence of abdominal distension, diarrhea, vomiting, and more than two symptoms in group A was lower, and the difference was statistically significant (*P* < 0.05). See Table 2 for details. 66.3% (65/98) of patients in group B achieved the expected energy goal within 72 hours, which was significantly higher than 37.1% (39/105) in group A (*P* < 0.05). The incidence of mechanical multiorgan dysfunction syndrome was lower in the B group than in the A group (*P* < 0.05) [7]. As shown in Figure 1, there was no statistical difference in mortality between the two groups (*P* > 0.05). See Table 3 for details.

The tolerance of critically ill patients to EN is not only affected by the EN regimen but also by gastrointestinal function and intra-abdominal pressure. The gastrointestinal tract of critically ill patients is an integral part of the body's organs; it is inevitably affected by different degrees of ischemia, hypoxia or perfusion injury, and systemic reactions [13]. Increased capillary permeability of intestinal mucosa will lead to intestinal wall edema and intestinal dysfunction. In turn, insufficient nutrition in intestinal cavity will lead to mucosal atrophy and reduced digestive juice secretion, the intestinal mucosa is atrophy, the blood supply of the intestinal wall is reduced, bacteria colonizes, and the inherent flora is destroyed. The permeability of the intestinal wall gradually increases, and bacteria and endotoxins are displaced; eventually, the intestinal barrier fails and the intra-abdominal pressure increases, making EN difficult to implement smoothly. The inspection and evaluation of EN tolerance are of great value for the control and rational application of EN complications. The study found that the higher the IAP of critically ill patients, the higher the incidence of abdominal distension or diarrhea. IAP level is one of the important factors leading to EN intolerance. The experimental group adjusted EN speed according to the IAP level, and the incidence of gastric retention, abdominal distension, and reflux was significantly lower than that of the control group, explaining that IAP monitoring should be carried out regularly in the implementation of EEN in critically ill patients; it can effectively prevent the occurrence of complications and increase the success rate of EEN implementation. The number of days of EN and ICU stay in the experimental group were less than those of the control group; it shows that IAP monitoring can effectively avoid further damage to the organ function of critically ill patients, improve patient prognosis, reduce hospitalization costs, and improve patient safety. The current research data show that the number of MODS cases and death cases in the experimental group are less than those in the control group; however, this study is still in progress, and more evidence needs to be collected to prove whether the incidence and mortality of patients with MODS are directly related to IAP levels [14].

Early EN support, on the one hand, can protect the intestinal mucosa, consolidate the intestinal barrier function, reduce bacterial translocation, and avoid elevated IAP; on the other hand, it can improve the degree of edema and exudation of organs and tissues and reduce the level of intra-abdominal pressure. IAP monitoring is of great significance to critically ill patients and can be used as a routine monitoring technology to popularize and apply [15]. However, there is no relevant knowledge of IAP monitoring in the current nursing textbooks, and many nurses have insufficient understanding of the importance of IAP. Fortunately, in recent years, the role of IAP monitoring in the treatment of critically ill patients has attracted more and more attention, such as raising the head of the bed, restlessness, coughing and sputum, and difficulty breathing, which may affect the level of intra-abdominal pressure, and too cold or hot water and fast perfusion rate may also increase intra-abdominal pressure. Therefore, before IAP monitoring, training must be strengthened and operating procedures must be standardized. When performing IAP measurement, nurses should try to eliminate external influence factors, including assisting the patient to take the supine position, removing the quilt, abdominal belt, proper sedation, accurately marking the zero point, controlling the temperature of the injected saline at 35∼37°C, and temporarily leaving the ventilator or suspending the use of PEEP when the condition permits. Replace the disposable connecting device every day, strictly aseptic operation, to prevent infection. When the measurement result does not match the condition, repeat the measurement 2 to 3 times and take the average value to ensure that the result is accurate and reliable.

## 4. Discussion

Severe patients tend to be malnourished, leading to prolonged mechanical ventilation, prolonged ICU hospitalization, increased infection complications, and increased mortality. The particular advantages of enteral nutrition in maintaining intestinal function and nutritional therapy have been widely recognized. As long as the gastrointestinal function allows, gastric residue monitoring is commonly used to guide enteral nutritional therapy. Multiple trauma (MT) is caused by machinery. Damage factors have great effects on the system state. The injury has severe pathophysiological changes and is life-threatening, often accompanied by abdominal infection, multiple organ dysfunction syndrome (plug-in) and other serious complications, and high mortality rate. Due to the rapid and complex changes of various injuries and diseases in ICU, the selection of simple and rapid organ function evaluation methods facilitates early diagnosis. Timely treatment and care are particularly important to improve the therapeutic efficacy in patients with MT [16].

ICU patients have a higher incidence of gastrointestinal dysfunction; acute gastrointestinal dysfunction caused by elevated abdominal pressure is a common clinical manifestation in critically ill patients. About 1/3 of mechanically ventilated patients develop abdominal hypertension, and gastrointestinal intolerance increases significantly during enteral nutrition therapy. The higher the intra-abdominal pressure, the higher the incidence of enteral nutrition intolerance; the level of intra-abdominal pressure is one of the important factors affecting the implementation of enteral nutrition in critically ill patients. Early administration of enteral nutrition in critically ill patients can prevent the increase in intra-abdominal pressure by protecting the intestinal mucosal barrier and can reduce intra-abdominal pressure by reducing gastrointestinal tissue edema [17].

In summary, compared to monitoring of residual gastric volume, it helps to improve the prognosis of patients [18, 19].

## 5. Conclusion

Reasonable enteral nutrition therapy can not only provide necessary nutrients but also it is safer and more feasible than intravenous nutrition support. However, early enteral nutrition support can also cause a series of poor tolerance. Control the amount and speed of enteral nutrition in critically ill patients in ICU by strengthening monitoring of intra-abdominal pressure and gastric residual volume; it can effectively prevent excessive retention in the stomach and cause abdominal distension and vomiting so that the patient reaches the target feeding amount as soon as possible; it is worthy of clinical application. In the observation of early enteral nutrition tolerance for patients in the intensive care unit, adopting intra-abdominal pressure combined with gastric residual monitoring method can significantly reduce the occurrence of adverse reactions in early enteral nutrition, reach the target feeding amount early to improve the patient's prognosis, shorten the time to adjust the enteral nutrition, and shorten the indwelling time in the ICU, and the effect is ideal.

## Figures and Tables

**Table 1:** Comparison of clinical data between the two groups of patients $\left(\bar{x}\pm s\right)$.

Group	Sex/case (%)		Age	Body mass index	Acute physiology and chronic health status score/point
	Men	Women			
Group A	57 (54.3)	48 (45.7)	66.32 ± 16.61	23.6 ± 2.1	22.9 ± 4.6
Group B	53 (54.1)	45 (45.9)	65.91 ± 15.36	23.3 ± 3.4	22.1 ± 4.3
*X* ^2^	0.001		0.181	0.156	1.023
*P*	>0.05		>0.05	>0.03	>0.13

**Table 2:** Comparison of enteral nutrition intolerance between the two groups of patients/cases (%).

Group	Abdominal distension	Diarrhea	Vomiting	2 or more symptoms
Group A	31 (29.5)	34 (32.4)	25 (23.8)	26 (24.8)
Group B	13 (13.3)	15 (15.3)	8 (8.2)	10 (10.2)
*X* ^2^	7.89	8.07	9.15	7.36
*P*	<0.05	<0.05	<0.05	<0.05

**Table 3:** Comparison of the number of days of enteral nutrition and ICU stay in the two groups of patients $\left(\bar{x}\pm s\right)$.

Group	Mechanical ventilation time (d)	Length of ICU stay per day	Ventilator-associated pneumonia/case (%)	Death during hospitalization/cases (%)
Group A	8.94 ± 2.35	9.19 ± 2.24	13 (12.4)	13 (12.4)
Group B	6.98 ± 1.45	7.38 ± 1.62	6 (6.1)	10 (10.2)
*X* ^2^	7.09	6.56	4.36	0.24
*P*	<0.05	<0.05	<0.05	<0.05

## Data Availability

The data used to support the findings of this study are available from the corresponding author upon request.
